# *Nlrp3* Deficiency Alleviates Lipopolysaccharide-Induced Acute Kidney Injury via Suppressing Renal Inflammation and Ferroptosis in Mice

**DOI:** 10.3390/biology12091188

**Published:** 2023-08-31

**Authors:** Zhilan Li, Xuan Wang, Yi Peng, Hongling Yin, Shenyi Yu, Weiru Zhang, Xin Ni

**Affiliations:** 1 Department of Rheumatology and Immunology, Xiangya Hospital, Central South University, Changsha 410008, China; 2 Department of General Medicine, Xiangya Hospital, Central South University, Changsha 410008, China; 3 Department of Pathology, Xiangya Hospital, Central South University, Changsha 410008, China; 4 Department of Rheumatology and Immunology, Zhuzhou Hospital Affiliated to Xiangya School of Medicine, Central South University, Zhuzhou 412007, China; 5 National Clinical Research Center for Geriatric Disorders, Xiangya Hospital, Central South University, Changsha 410008, China; 6 International Collaborative Research Center for Medical Metabolomics, Xiangya Hospital, Central South University, Changsha 410008, China

**Keywords:** NLRP3, acute kidney injury, sepsis, inflammation, ferroptosis

## Abstract

**Simple Summary:**

Acute kidney injury is a common and severe group of clinical syndromes with numerous causes, including sepsis. The molecular mechanisms underlying sepsis-associated acute kidney injury development remain largely unknown, which explains the limitations of current clinical strategies. The NLRP3 inflammasome, the most widely researched inflammasome in the kidney, is crucial in the pathogenesis of sepsis and acute kidney injury. Herein, we sought to determine the expression of NLRP3 in patients with sepsis-associated acute kidney injury and investigate the significance and mechanisms of NLRP3 involvement. According to our data, patients with sepsis-associated acute kidney injury had upregulated NLRP3 expression in their kidneys. In addition, *Nlrp3* deficiency strikingly attenuated sepsis-associated acute kidney injury. Mechanically, we found that *Nlrp3* knockout reduced inflammation, reversed metabolic pathway changes, and decreased ferroptosis in the mouse kidneys. These findings indicate that *Nlrp3* deficiency ameliorates sepsis-associated acute kidney injury via suppressing renal inflammation and ferroptosis and that substance metabolism modulation may be of importance for NLRP3 functioning. This sheds new light on the mechanisms of NLRP3 involvement in sepsis-associated acute kidney injury and provides further evidence for considering NLRP3 as a therapeutic target.

**Abstract:**

The nucleotide-binding oligomerization domain-like receptor protein 3 (NLRP3) inflammasome is a vital component of many inflammatory responses. Here, we intended to investigate the involvement of NLRP3 in lipopolysaccharide (LPS)-induced sepsis-associated acute kidney injury (S-AKI) and explore its mechanisms. For the first time, we validated elevated NLRP3 expression in the renal tissues of S-AKI patients by immunohistochemistry analysis. Through LPS injection in both wild-type and *Nlrp3^−/−^* mice, a S-AKI model was developed. It was found that LPS-induced kidney injury, including an abnormal morphology in a histological examination, abnormal renal function in a laboratory examination, and an increase in the expression of AKI biomarkers, was dramatically reversed in *Nlrp3*-deficient mice. *Nlrp3* deletion alleviated renal inflammation, as evidenced by the suppression of the expression of pro-inflammatory cytokines and chemokines. A combinative analysis of RNA sequencing and the FerrDb V2 database showed that *Nlrp3* knockout regulated multiple metabolism pathways and ferroptosis in LPS-induced S-AKI. Further qPCR coupled with Prussian blue staining demonstrated that *Nlrp3* knockout inhibited murine renal ferroptosis, indicating a novel mechanism involving S-AKI pathogenesis by NLRP3. Altogether, the aforementioned findings suggest that *Nlrp3* deficiency alleviates LPS-induced S-AKI by reducing renal inflammation and ferroptosis. Our data highlight that NLRP3 is a potential therapeutic target for S-AKI.

## 1. Introduction

Sepsis-associated acute kidney injury (S-AKI), one of the most prevalent causes of AKI, ranks first among patients with AKI in the intensive care unit [1]. It has been reported that more than 60% of patients with septic shock can develop early AKI, indicating that AKI is a common disorder in sepsis [2]. S-AKI is frequently linked to higher in-hospital mortality, increased hospitalization duration, renal failure, and the development of chronic kidney disease (CKD) [1,2,3]. The clinical strategy is currently limited to renal replacement therapy without any other particularly effective therapies [4]. Such circumstances are mainly attributed to the fact that the molecular mechanisms governing S-AKI pathogenesis are not fully understood, although the intensive inflammatory responses in renal tissues are the central events in S-AKI pathogenesis.

The nucleotide-binding oligomerization domain-like receptor protein 3 (NLRP3) is a cytoplasmic pattern-recognition receptor (PRR) that undergoes self-oligomerization, recruits the apoptosis-associated speck-like protein containing a caspase recruitment domain (ASC), and then combines with pro-caspase-1 to form the NLRP3/ASC/pro-caspase-1 protein complex, known as the NLRP3 inflammasome, in response to various stimuli such as pathogen-associated molecular patterns (PAMPs) and damage-associated molecular patterns (DAMPs) [5]. The subsequent activation of pro-caspase-1 triggered by assembled NLRP3 inflammasome leads to the maturation and release of interleukin-1β (IL-1β) and interleukin-18 (IL-18), and to gasdermin D (GSDMD)-dependent pyroptosis [6]. Increasing research has suggested that the NLRP3 inflammasome is activated in renal tissues and thereby participates in the pathophysiology of S-AKI [7,8,9,10,11,12]. Cao et al. [13] reported that *Nlrp3* knockout could alleviate mouse S-AKI induced by cecal ligation and puncture (CLP) and reduce renal neutrophil infiltration, as well as IL-1β and IL-18 levels. In fact, in addition to inflammatory responses, the pathophysiology of S-AKI has also been linked to other mechanisms, such as impaired microcirculation and the metabolic rewiring of tubular epithelial cells [14]. Thus, the mechanism through which NLRP3 promotes S-AKI development is still largely unknown.

Although NLRP3 inflammasome activation has been found in S-AKI in animal models, its expression in human renal tissues with S-AKI has not been reported according to our knowledge. Here, we firstly examined NLRP3 expression in the human kidneys of patients with S-AKI. Sepsis is well documented to frequently be related to infection with gram-negative bacteria [15]. Thus, lipopolysaccharide (LPS)-induced AKI is a common model for exploring the mechanism of S-AKI because LPS is a component of gram-negative bacteria. In order to figure out the molecular mechanism underlying S-AKI associated with the NLRP3 inflammasome, we investigated the molecular network affected by LPS treatment and *Nlrp3* deletion in the mouse renal tissues using RNA sequencing in combination with the FerrDb V2 database. We revealed that ferroptosis was involved in LPS-induced AKI, which could be alleviated by *Nlrp3* deficiency.

## 2. Materials and Methods

### 2.1. Human Kidney Samples

The Xiangya Hospital of Central South University at Changsha provided the human kidney samples used in this study. Patients with clinically diagnosed sepsis and renal biopsy or nephrectomy specimens showing renal injury were selected as the S-AKI group (n = 4). Patients with minimally changed renal biopsies and without urinary tract infections were selected as the control group (n = 5). Patients’ clinical data before renal biopsy or nephrectomy were also collected. The clinical data are listed in Table 1. The Clinical Medical Ethics Committee of Xiangya Hospital of Central South University approved the research protocol after receiving informed consent from the participants (approval number: 2022020606).

### 2.2. Animals and S-AKI Model

Global *Nlrp3* knockout (*Nlrp3^−/−^*) mice were established by using Dppa3-Cre tool mice expressing Cre recombinase in most tissues and *Nlrp3*-loxP mice on a C57BL/6 background purchased from the Nanjing Biomedical Research Institute of Nanjing University. *Nlrp3^−/−^* mice were genotyped in accordance with the recommended protocol. Throughout the study, mice were kept and maintained in a specific pathogen-free environment in the animal care facility of the Xiangya Medical College of Central South University at Changsha, where they had unlimited access to water and food. The Experimental Animal Welfare Ethics Committee of Central South University conducted a thorough examination and granted approval for all protocols pertaining to the use of animals in this study (approval number: CSU-2022-01-0111).

Wild-type (WT) and *Nlrp3^−/−^* male mice (22–29 g) were grouped randomly. The S-AKI model was constructed by injecting intraperitoneally (i.p.) with 5 mg/kg of LPS (*E. coli* O111:B4, Sigma, St. Louis, MO, USA) dissolved in normal saline (NS) (n = 9–11 per group) [8]. Mice in the control groups were administered with an equal volume of NS (n = 10–11 per group). After 24 h, all mice were anesthetized, and their eyeball blood and kidneys were collected. For subsequent examination, mouse serum and renal tissues were stored at −80 °C.

### 2.3. Histological Assessment

To evaluate renal pathological changes, mouse renal tissues fixed in 4% paraformaldehyde were embedded in paraffin, and sections cut from the paraffin were stained with hematoxylin and eosin (HE) or periodic ac-id-Schiff (PAS) at a thickness of 4 μm. Images were obtained under a light microscope (Leica, Wetzlar, Germany). The tubulointerstitial injury score was calculated for each HE slide as previously described [17].

### 2.4. Prussian Blue Staining

Paraffin sections of mouse renal tissues were dewaxed and hydrated. Tissue iron detection was then performed using a Prussian blue staining kit (G1029, Servicebio, Wuhan, China) as directed by the manufacturer. Pieces were viewed and photographed using a light microscope (Olympus, Tokyo, Japan).

### 2.5. Immunohistochemistry (IHC)

Paraffin slides of human renal tissues were dewaxed, hydrated, and microwaved in EDTA (pH 9.0) for 10 min to perform antigen retrieval. Next, the slides underwent a 30 min exposure to 3% hydrogen peroxide, followed by a 1 h blocking step at 37 °C using goat serum. After overnight incubation with the antibody against NLRP3 (1:800, PA5-79740, Thermo Fisher Scientific, Carlsbad, CA, USA) at 4 °C, the slides subsequently underwent a 45 min incubation at 37 °C with a horseradish peroxidase (HRP)-coupled secondary antibody. This was followed by staining with DAB and hematoxylin. Finally, pieces were viewed and photographed using a light microscope (Olympus, Tokyo, Japan). Five randomly chosen fields at 200× magnification in each slice were analyzed semi-quantitatively using ImageJ software (NIH, Bethesda, MD, USA). The average proportion of stained area per kidney was used as the expression level of NLRP3.

### 2.6. Real-Time Quantitative PCR (qPCR)

Using a Trizol reagent (Life Technologies, Gaithersburg, MD, USA), total RNA was isolated from mouse renal tissues. Afterwards, 2 μg of RNA was used to carry out reverse transcription, employing a cDNA synthesis kit (TransGen Biotech, Beijing, China). The cDNA was subsequently diluted 10 times before being used to conduct quantitative PCR in accordance with the suggested procedure in the QuantStudio Real-Time PCR detection biosystem (Thermo Fisher Scientific, Carlsbad, CA, USA) with a qPCR Mix (AG11718, Accurate Biotechnology (Hunan) Co., Ltd., Changsha, China). β-actin was used as a reference gene to determine the target gene expression. Table 2 contains a list of all mouse primer sequences (Sangon Biotech Co., Shanghai, China) utilized in the present study.

### 2.7. Laboratory Examination

Serum creatinine and urea levels were measured in the Department of Clinical Laboratory, Xiangya Hospital, with an automated biochemical analysis instrument (AU5400, Olympus, Tokyo, Japan).

### 2.8. RNA Sequencing and Bioinformatics Analysis

In the WT + NS, WT + LPS, and *Nlrp3^−/−^* + LPS groups, total RNA was isolated from the mouse kidneys (n = 3 per group) with a Trizol reagent (Life Technologies, Gaithersburg, MD, USA). Next, the library construction was performed after evaluating RNA integrity, followed by RNA sequencing on the Illumina NovaSeq 6000 platform (Novogene, Beijing, China). In order to reduce the false discovery rate, a necessary adjustment to *p*-values was made utilizing Benjamini and Hochberg’s method. Along with |log_2_FoldChange| > 1, an adjusted *p*-value (p.adjust) < 0.05 found by DESeq2 was the criteria for identifying differentially expressed genes (DEGs). The volcano maps, Venn diagrams, and heatmaps of DEGs were plotted by https://www.bioinformatics.com.cn (accessed on 19 April 2023), an online platform for data analysis and visualization. Gene Ontology (GO) and Kyoto Encyclopedia of Genes and Genomes (KEGG) pathway enrichment analyses of DEGs were conducted on the Metascape website [18] and visualized using https://www.bioinformatics.com.cn (accessed on 19 April 2023).

The ferroptosis-related genes, including driver, suppressor, and marker genes, were obtained from the FerrDb V2 database [19], which was also used to conduct Gene Set Enrichment Analysis (GSEA). The intersection of DEGs in RNA sequencing and ferroptosis-related genes was defined as ferroptosis-related DEGs (FerDEGs), which was visualized by the Venn diagram.

### 2.9. Statistical Analysis

The data are presented as mean ± SEM. Statistical analysis was conducted utilizing GraphPad Prism 8.0.1 software (GraphPad, San Diego, CA, USA). When comparing two groups, an unpaired *t*-test and chi-squared test were used for continuous and categorical variables, respectively; when comparing more than two groups, one-way analysis of variance (ANOVA) with Turkey’s test for multiple comparisons was applied. *p* < 0.05 was considered statistically significant.

## 3. Results

### 3.1. Patients with S-AKI Have an Elevated Level of NLRP3 Expression in the Kidneys

**Figure 1:**
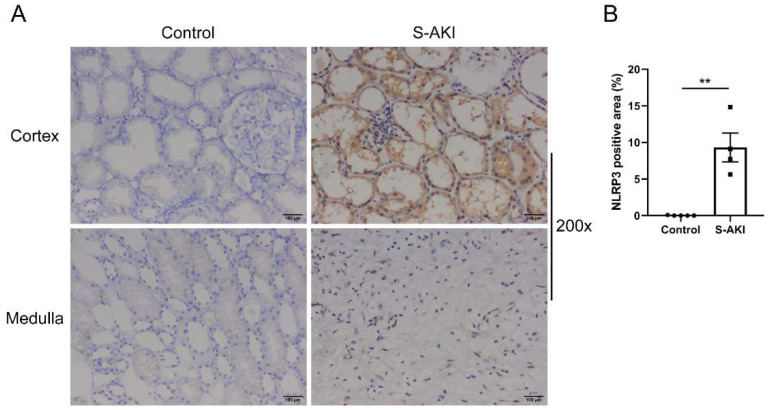
NLRP3 was upregulated in the renal tissues of patients with S-AKI. (**A**) Representative IHC staining images of NLRP3 from human kidney paraffin sections (200×). Control was minimally changed kidney. Scale bar = 100 μm. (**B**) Semi-quantification of IHC staining of NLRP3 in S-AKI patients (n = 4) vs. the control group (n = 5). The data are presented as mean ± SEM. ** *p* < 0.01. NLRP3: nucleotide-binding oligomerization domain-like receptor protein 3; S-AKI: sepsis-associated acute kidney injury; IHC: immunohistochemistry.

To test the expression of NLRP3 in S-AKI, we recruited four S-AKI patients and five control patients, who had undergone renal biopsy or nephrectomy. As shown in Figure 1A, NLRP3-positive staining was identified in renal tubular epithelial cells and interstitial infiltrating inflammatory cells. The level of NLRP3 expression exhibited a significant increase in the kidneys of S-AKI patients compared with controls (Figure 1B).

### 3.2. Nlrp3 Deficiency Relieves LPS-Induced Renal Injury in Mice

**Figure 2:**
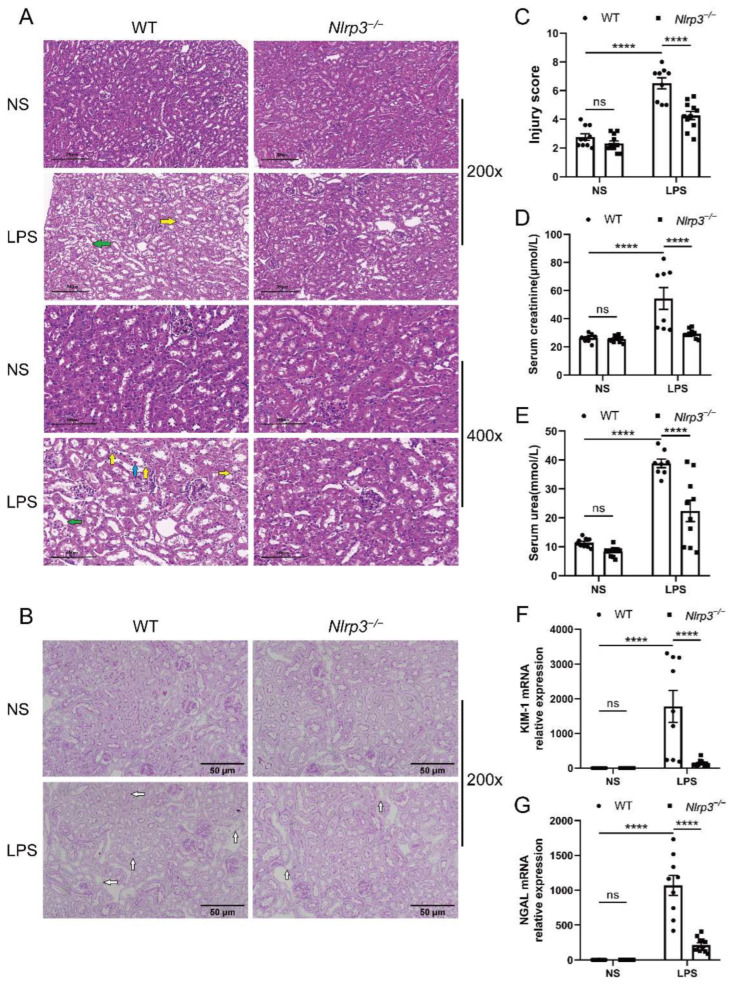
*Nlrp3* deletion alleviated renal pathological damage and ameliorated renal function in LPS-induced S-AKI in vivo. WT and *Nlrp3^−/−^* mice were injected intraperitoneally with LPS or saline and sacrificed to collect serum and renal tissues after 24 h. (**A**) Representative HE staining images of mouse renal tissues (200× and 400×). The WT + LPS group presented obvious tubular cell vacuolization (indicated by the yellow arrows), interstitial edema (indicated by the green arrows), and tubular cell sloughing off into the lumen (indicated by the blue arrow). Scale bar = 200 μm at 200× magnification, and scale bar = 100 μm at 400× magnification. (**B**) Representative PAS staining images of mouse renal tissues (200×). The *Nlrp3^−/−^* + LPS group had a less obvious loss of the brush border (indicated by the white arrows) compared with the WT + LPS group. Scale bar = 50 μm. (**C**) The tubulointerstitial injury score of different groups of mice. (**D**,**E**) Serum creatinine and urea levels of different groups of mice. (**F**,**G**) Renal KIM-1 and NGAL mRNA levels of mice from different groups measured by qPCR. The data are presented as mean ± SEM (n = 8–11 per group). **** *p* < 0.0001. ns: no significance. *Nlrp3*: nucleotide-binding oligomerization domain-like receptor protein 3; S-AKI: sepsis-associated acute kidney injury; WT: wild-type; *Nlrp3^−/−^*: global *Nlrp3* knockout; NS: normal saline; LPS: lipopolysaccharide; HE: hematoxylin and eosin; PAS: periodic acid-Schiff; KIM-1: kidney injury molecule 1; NGAL: neutrophil gelatinase-associated lipocalin; qPCR: real-time quantitative PCR.

The S-AKI model was established through LPS treatment in age-matched male WT and *Nlrp3^−/−^* mice, and renal injury was assessed. WT mice with LPS treatment presented different degrees of pathological damage compared with the mice with vehicle treatment, as evidenced by tubular cell vacuolization or sloughing, tubular dilation or atrophy, tubular cast formation, interstitial edema, and interstitial infiltration of a few inflammatory cells (Figure 2A). PAS staining showed that WT mice had a more obvious loss of the brush border after exposure to LPS (Figure 2B). Meanwhile, the semi-quantitative tubulointerstitial injury score showcased a significant increase in WT mice following LPS administration (Figure 2C). In contrast, *Nlrp3*-deficient mice with LPS treatment displayed less intensity of injury, as evidenced by diminished interstitial edema, decreased tubular cell vacuolization, and reduced brush border loss compared to WT mice with LPS treatment (Figure 2A,B). Likewise, *Nlrp3*-deficient mice had a much lower injury score after LPS treatment compared with WT mice (Figure 2C).

Laboratory examination showed that creatinine and urea levels in the circulation were considerably higher in WT mice after LPS administration (Figure 2D,E). *Nlrp3* deficiency markedly reversed the increase in serum concentrations of creatinine and urea induced by LPS treatment (Figure 2D,E).

In consistence with the histological changes and laboratory examination, WT mice treated with LPS exhibited dramatically increased mRNA levels of AKI biomarkers, kidney injury molecule 1 (KIM-1) and neutrophil gelatinase-associated lipocalin (NGAL), in the renal tissues (Figure 2F,G). Increased KIM-1 and NGAL expression induced by LPS treatment were notably reversed in *Nlrp3*-deficient mice (Figure 2F,G). Collectively, these results demonstrate that *Nlrp3* deletion relieved S-AKI induced by LPS in mice.

### 3.3. Nlrp3 Deficiency Alleviates Renal Inflammation in S-AKI Mice Induced by LPS

**Figure 3:**
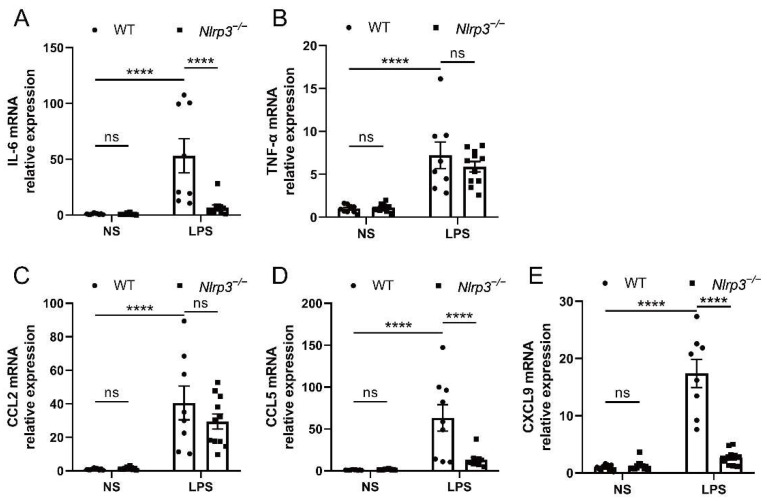
*Nlrp3* deletion suppressed the mRNA expression of pro-inflammatory cytokines and chemokines in the mouse renal tissues. (**A**,**B**) The mRNA levels of IL-6 and TNF-α in the whole kidney homogenates of mice measured by qPCR. (**C**–**E**) The mRNA levels of pro-inflammatory chemokines, CCL2, CCL5, and CXCL9, in the whole kidney homogenates of mice measured by qPCR. The data are presented as mean ± SEM (n = 9–11 per group). **** *p* < 0.0001. ns: no significance. *Nlrp3*: nucleotide-binding oligomerization domain-like receptor protein 3; WT: wild-type; *Nlrp3^−/−^*: global *Nlrp3* knockout; NS: normal saline; LPS: lipopolysaccharide; IL-6: interleukin-6; TNF-α: tumor necrosis factor-α; CCL2: C-C motif chemokine ligand 2/monocyte chemoattractant protein-1 (MCP-1); CCL5: C-C motif chemokine ligand 5; CXCL9: C-X-C motif chemokine ligand 9; qPCR: real-time quantitative PCR.

Since the NLRP3 inflammasome has been recognized as a key mediator of the renal inflammatory response, we investigated the inflammatory factors in the follow-up experiments. The results showed that WT mice with LPS administration had elevated mRNA expression of interleukin-6 (IL-6), tumor necrosis factor-α (TNF-α), C-C motif chemokine ligand 2/monocyte chemoattractant protein-1 (CCL2/MCP-1), C-C motif chemokine ligand 5 (CCL5), and C-X-C motif chemokine ligand 9 (CXCL9) in the renal tissues, as compared with the mice with saline treatment (Figure 3A–E). On the contrary, in *Nlrp3*-deficient mice, the LPS-induced increase in mRNA levels of IL-6, CCL5, and CXCL9 was significantly reversed. Similarly, the mRNA expression of TNF-α and CCL2 showed a downward trend despite no significant difference in *Nlrp3*-deficient mice following LPS treatment in comparison with WT mice receiving LPS treatment. These data suggest that *Nlrp3* deletion reduced renal inflammation in S-AKI mice induced by LPS.

### 3.4. Nlrp3 Deficiency Affects Multiple Metabolic Pathways and Ferroptosis Pathways in S-AKI In Vivo

**Figure 4:**
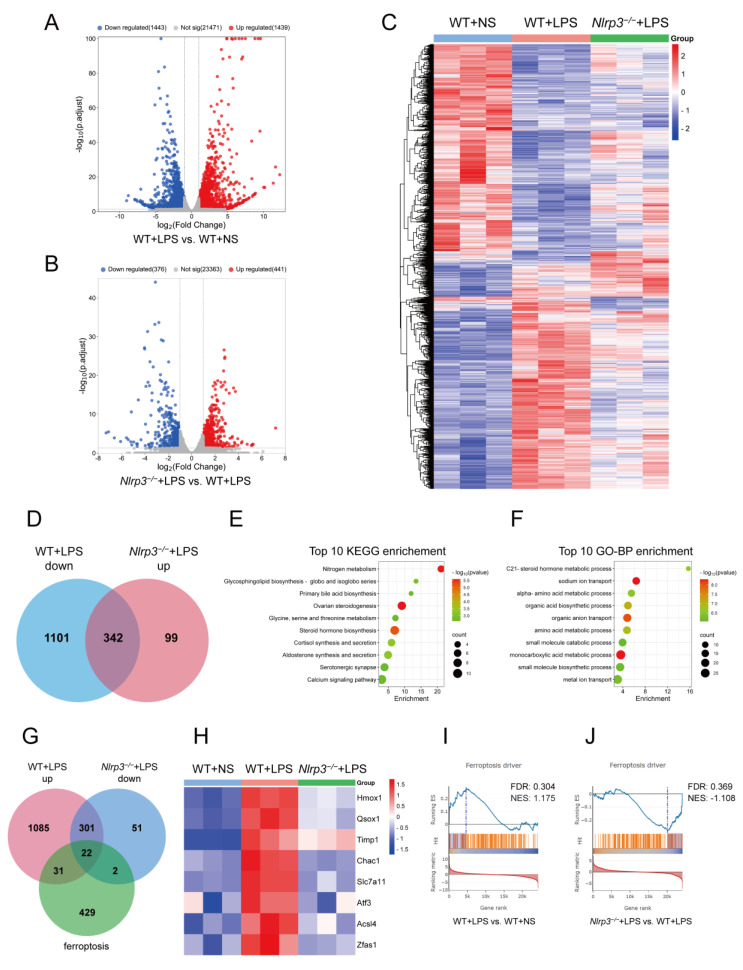
A combinative analysis of RNA sequencing of mouse renal tissues and the FerrDb V2 database. Renal tissues of mice in WT + NS, WT + LPS, and *Nlrp3^−/−^* + LPS groups were subjected to RNA sequencing analysis (n = 3 per group). (**A**) Volcano plot of DEGs between the WT + LPS group and the WT + NS group. (**B**) Volcano plot of DEGs between the *Nlrp3^−/−^* + LPS group and the WT + LPS group. The upregulated genes are denoted by right-hand red dots, the downregulated genes are denoted by left-hand blue dots, and insignificant genes are denoted by grey dots. (**C**) Clustering heatmap of DEGs among the WT + NS, WT + LPS, and *Nlrp3^−/−^* + LPS groups. Red indicates upregulation, blue indicates downregulation, and white indicates no difference. (**D**) The Venn diagram shows the intersection of genes downregulated in WT + LPS vs. WT + NS (WT + LPS down) and genes upregulated in *Nlrp3^−/−^* + LPS vs. WT + LPS (*Nlrp3^−/−^* + LPS up). (**E**,**F**) Top 10 pathways in KEGG and GO-BP enrichment analyses on the intersection of WT + LPS down genes and *Nlrp3^−/−^* + LPS up genes. (**G**) The Venn diagram shows the intersection of genes upregulated in WT + LPS vs. WT + NS (WT + LPS up), genes downregulated in *Nlrp3^−/−^* + LPS vs. WT + LPS (*Nlrp3^−/−^* + LPS down), and 484 ferroptosis-related genes downloaded from the FerrDb V2 database (ferroptosis). (**H**) Heatmap of eight ferroptosis driver DEGs according to RNA sequencing data. (**I**,**J**) GSEA analysis of ferroptosis driver between the WT + LPS group and the WT + NS group (**I**) and between the *Nlrp3^−/−^* + LPS group and the WT + LPS group (**J**) performed in the FerrDb V2 database. WT: wild-type; *Nlrp3^−/−^*: global *Nlrp3* knockout; NS: normal saline; LPS: lipopolysaccharide; DEGs: differentially expressed genes; KEGG: Kyoto Encyclopedia of Genes and Genomes; GO-BP: Gene Ontology-Biological Process; GSEA: Gene Set Enrichment Analysis; p.adjust: adjusted *p*-value; Not sig: not significant; Hmox1: heme oxygenase 1; Qsox1: quiescin Q6 sulfhydryl oxidase 1; Timp1: tissue inhibitor of metalloproteinase 1; Chac1: ChaC, cation transport regulator 1; Slc7a11: solute carrier family 7 (cationic amino acid transporter, y + system), member 11; Atf3: activating transcription factor 3; Acsl4: Acyl-CoA synthetase long-chain family member 4; Zfas1: zinc finger, NFX1-type containing 1, antisense RNA 1. FDR: false discovery rate; NES: normalized enrichment score.

To investigate the underlying mechanisms of *Nlrp3* knockout attenuating LPS-induced S-AKI, the renal cortex of mice from the WT + NS, WT + LPS, and *Nlrp3^−/−^* + LPS groups was subjected to RNA sequencing. As shown in Figure 4A, the RNA sequencing analysis revealed 1439 upregulated genes and 1443 downregulated genes in the renal tissues of WT mice following LPS administration. *Nlrp3^−/−^* mice with LPS treatment displayed 441 upregulated genes and 376 downregulated genes compared to the WT mice following LPS treatment (Figure 4B). The heatmap of the DEGs among the three groups is shown in Figure 4C. Next, in order to determine which genes with altered expression after LPS treatment were reversed by *Nlrp3* knockout, we considered the intersection of genes downregulated in WT mice after LPS administration (WT + LPS down) and upregulated in *Nlrp3^−/−^* mice treated with LPS (*Nlrp3^−/−^* + LPS up), and 342 genes were screened out (Figure 4D). We conducted KEGG pathway and GO-Biological Process (BP) enrichment analyses on these 342 genes, showing that ion transport and synthesis and metabolism processes of multiple substances, including nitrogen, lipids, bile acid, steroid hormone, amino acids, small molecules, and monocarboxylic acid, were prominently enriched, with nitrogen metabolism and the steroid hormone metabolic process ranking first (Figure 4E,F). These enriched pathways are not isolated but closely related; for example, amino acids are one of the main sources and flow destinations of nitrogen in cells, cortisol and aldosterone are steroid hormones, and aldosterone governs sodium ion transport. The results imply the impact of *Nlrp3* knockout on renal metabolism in S-AKI caused by LPS.

Furthermore, since ferroptosis is associated with ferric ion transport and fatty acid metabolism and has been proven involved in the development of S-AKI [20,21], we then extracted 484 ferroptosis-related genes (ferroptosis) from the FerrDb V2 database and intersected them with genes upregulated in WT mice after LPS administration (WT + LPS up) and genes downregulated in *Nlrp3^−/−^* mice treated with LPS (*Nlrp3^−/−^* + LPS down). As a result, we obtained 22 ferroptosis-related DEGs (FerDEGs) (Figure 4G and Table S1). Eight of these 22 FerDEGs are driver genes, including heme oxygenase 1 (Hmox1), quiescin Q6 sulfhydryl oxidase 1 (Qsox1), tissue inhibitor of metalloproteinase 1 (Timp1), ChaC, cation transport regulator 1 (Chac1), solute carrier family 7 (cationic amino acid transporter, y + system), member 11 (Slc7a11), activating transcription factor 3 (Atf3), acyl-CoA synthetase long-chain family member 4 (Acsl4), and zinc finger, NFX1-type containing 1, antisense RNA 1 (Zfas1) (Figure 4H). Additionally, GSEA analysis was performed in the FerrDb V2 database, showing that the ferroptosis driver was upregulated in WT mice after LPS administration and downregulated in *Nlrp3^−/−^* mice treated with LPS (Figure 4I,J). The results indicate that *Nlrp3* knockout affected renal ferroptosis pathways in the LPS-induced S-AKI model.

### 3.5. Nlrp3 Knockout Attenuates Renal Ferroptosis in S-AKI Mice Induced by LPS

**Figure 5:**
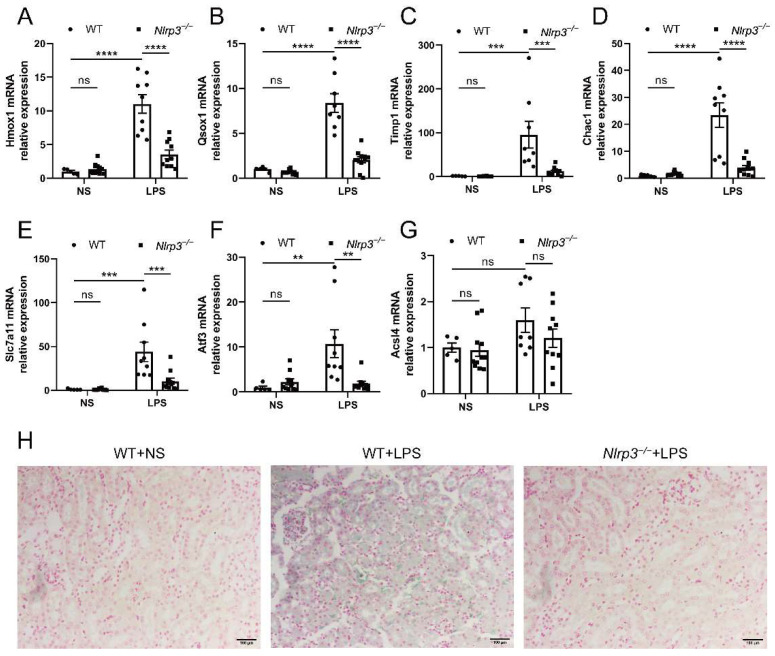
*Nlrp3* deletion reduced renal ferroptosis in LPS-induced S-AKI in vivo. (**A**–**G**) The mRNA levels of Hmox1, Qsox1, Timp1, Chac1, Slc7a11, Atf3, and Acsl4 in the whole kidney homogenates measured by qPCR. (**H**) Representative Prussian blue staining images of mouse renal tissues (200×). The blue-green color represents ferritin deposition, showing that the *Nlrp3^−/−^* + LPS group had fewer iron-containing vesicles in the kidney than the WT + LPS group. Scale bar = 100 μm. The data are presented as mean ± SEM (n = 5–11 per group). ** *p* < 0.01, *** *p* < 0.001, **** *p* < 0.0001. ns: no significance. *Nlrp3*: nucleotide-binding oligomerization domain-like receptor protein 3; WT: wild-type; *Nlrp3^−/−^*: global *Nlrp3* knockout; NS: normal saline; LPS: lipopolysaccharide; Hmox1: heme oxygenase 1; Qsox1: quiescin Q6 sulfhydryl oxidase 1; Timp1: tissue inhibitor of metalloproteinase 1; Chac1: ChaC, cation transport regulator 1; Slc7a11: solute carrier family 7 (cationic amino acid transporter, y + system), member 11; Atf3: activating transcription factor 3; Acsl4: Acyl-CoA synthetase long-chain family member 4; qPCR: real-time quantitative PCR.

In order to validate that *Nlrp3* knockout regulated ferroptosis in S-AKI caused by LPS, the expression of ferroptosis driver DEGs and ferritin deposition in the mouse renal tissues were measured. As a note, Zfas1 expression was not measured, as no suitable primers were found. As shown in Figure 5A–G, while Acsl4 expression was not significantly different, the mRNA levels of the other six ferroptosis driver genes (Hmox1, Qsox1, Timp1, Chac1, Slc7a11, and Atf3) were significantly increased in response to LPS, which were prevented by *Nlrp3* deficiency. Similarly, Prussian blue staining showcased obvious ferritin deposition in renal tissues of WT mice upon LPS treatment, mostly in renal tubular epithelial cells and interstitial infiltrating cells (Figure 5H), which was where NLRP3 was found in the kidneys of S-AKI patients. Ferritin deposition was robustly reduced in *Nlrp3* knockout mice with LPS treatment compared with WT mice with LPS treatment (Figure 5H). These results suggest that *Nlrp3* knockout reduced renal ferroptosis in S-AKI mice induced by LPS.

## 4. Discussion

Here, we verified the upregulation of NLRP3 expression in the kidneys of S-AKI patients. Additionally, we found that *Nlrp3* knockout alleviated renal injury and inflammation in mice exposed to LPS, confirming the role of NLRP3 in LPS-induced S-AKI. Moreover, *Nlrp3* deficiency reversed multiple metabolic pathways and decreased ferroptosis in renal tissues of the LPS-induced S-AKI model. These findings shed fresh perspectives on the mechanisms of NLRP3 involvement in S-AKI progression.

The most popularly employed animal models of S-AKI include LPS injection and CLP. Although the clinical relevance of the LPS model is less than that of CLP, it has the advantages of convenience, good repeatability, and little influence from the technology of operators [22]. In addition, gram-negative bacteria containing LPS are the main pathogens of sepsis [15], and injecting low-dose LPS into healthy human volunteers induces pathophysiologic alterations similar to those observed in patients with sepsis [22], which indicates the value of the LPS model in the research of sepsis. In this study, a relatively small dosage of LPS (5 mg/kg) was injected intraperitoneally over the course of 24 h to construct a non-lethal and mildly injured mouse model of S-AKI [8].

The NLRP3 inflammasome is widely recognized as a classical and extensively researched inflammasome in the kidney. As an initial member of the inflammasome activation, NLRP3 is involved in the pathophysiology of numerous renal diseases in both inflammasome-dependent and inflammasome-independent ways [23]. While prior research has demonstrated elevated NLRP3 in the kidneys of S-AKI models as well as in the serum of patients with sepsis and the significant involvement of the NLRP3 inflammasome in the progression of S-AKI [8,13,24,25], there is currently no reported study on the expression of NLRP3 in renal tissues of S-AKI patients. Furthermore, the evidence of whether *Nlrp3* deficiency can protect against S-AKI induced by LPS in vivo is very limited. Through the utilization of human renal biopsy or nephrectomy slices, we verified for the first time enhanced NLRP3 expression in the kidneys of patients with S-AKI, which provides preliminary support for further translational research. However, we did not validate the upregulation of NLRP3 in the renal tissues of S-AKI mice. On the whole, our results showed that the deletion of *Nlrp3* strikingly alleviated renal pathological damage and improved renal function, as previously reported in a CLP model [13]. We also investigated the expression of AKI biomarkers, KIM-1 and NGAL, which exhibit greater sensitivity and earlier changes than serum creatinine when AKI occurs [26]. Moreover, *Nlrp3* deletion had no impact on the kidneys of mice without LPS treatment in our study. These results strongly illustrate that *Nlrp3* knockout ameliorated mouse renal injury caused by LPS, providing further evidence for the significance of NLRP3 in the development of S-AKI.

Excessive inflammatory responses characterized by inflammatory cell infiltration and cytokine storms are the key circumstances that lead to S-AKI progression [14]. Previous studies have proven that NLRP3 facilitates the inflammatory response in sepsis [13,27]. Although it is known that inflammasome activation is an important pathway for NLRP3 to mediate the inflammatory response, this study was aimed to investigate the mechanisms through which NLRP3 promotes S-AKI development other than the inflammasome pathway. Thus, we assessed the expression of other inflammatory factors instead of IL-1β and IL-18. IL-6 and TNF-α are classical pro-inflammatory cytokines, which were reduced by *Nlrp3* deletion in LPS-induced S-AKI mice in our study, though the decrease in TNF-α was not significant. We also found that the LPS-induced expression of several chemokines, including CCL2, CCL5, and CXCL9, was inhibited by *Nlrp3* knockout. CCL2, also known as monocyte chemoattractant protein-1 (MCP-1), is a pivotal mediator of monocyte and macrophage migration and assumes an essential function in the pathological process of renal diseases related to inflammation and AKI [28,29]. A recent study showed that S-AKI caused by LPS in mice was significantly alleviated when CCL2 was specifically deficient in renal proximal tubules [30]. However, in our study, the CCL2 mRNA level did not significantly decline after *Nlrp3* knockout, which was probably related to the large individual differences in the WT + LPS group. Another bioinformatics study showed that CXCL9 is one of the key genes involved in multiple organ injuries caused by LPS in mice [31]. While the renal interstitial infiltration was relatively mild due to the low dose of LPS, the results of inflammatory factors suggest that *Nlrp3* deletion attenuated renal inflammation.

Metabolic rewiring is currently recognized as one of the main mechanisms of S-AKI development [14]. The kidney has one of the highest metabolic rates among all organs, primarily due to proximal tubular epithelial cells [32]. In the pathophysiological process of S-AKI, for the purpose of maintaining cell survival, oxidative phosphorylation and fatty acid oxidation switch to anaerobic metabolism, accompanied by a halt in tubular ion transport, which could lead to organ dysfunction [33]. Metabolic pathways, including metabolites and enzymes, can activate and regulate the NLRP3 inflammasome [34]. In turn, whether NLRP3 can regulate substance and energy metabolism is not fully understood. Recent studies showed that two different NLRP3 inhibitors reversed the upregulation of glycolysis in a model of LPS-induced acute neuroinflammation and affected cerebral glucose and lipid metabolism in Alzheimer’s disease model mice [35,36]. Another study reported lipid metabolism changes in the liver of *Nlrp3* knockout mice [37]. Importantly, our study found that *Nlrp3* knockout reversed the downregulation in renal metabolic pathways induced by LPS, including ion transport and the metabolism of multiple substances, with nitrogen and steroid hormone metabolisms ranking first among the KEGG and GO-BP enrichment pathways, respectively. Nitrogen is necessary for the biosynthesis of nucleotides, amino acids, glutathione, nitric oxide, and many other key biological compounds, so nitrogen metabolism plays a fundamental role in cell growth and proliferation and modulating immune cell function [38]. On the other hand, steroid hormones, predominantly produced in the adrenal cortex, especially cortisol, which has the biological functions of modulating metabolism and immune response under stress, are critical for an organism’s defense against sepsis [39,40]. In the present study, we found that cortisol and aldosterone synthesis and secretion pathways were downregulated after LPS treatment and that *Nlrp3* knockout reversed them. In fact, we have previously demonstrated that the same LPS administration (5 mg/kg, i.p., 24 h) triggered adrenocortical hyporesponsiveness to adrenocorticotropic hormone (ACTH) stimulation in mice [41,42], which can be used as a validation of the enrichment analysis results in this study. It has been reported that NLRP3 expression is elevated in the adrenal glands of mice exposed to LPS [43]. Altogether, it implied that *Nlrp3* knockout may partly mitigate LPS-induced S-AKI through restoring adrenocortical function. Of note, the finding that *Nlrp3* knockout regulates renal metabolism-related pathways, including steroid hormone metabolism, is an innovation of our study, which supports that we should pay attention to the adrenal function of patients with S-AKI in clinical practice and that, in addition to energy metabolic rewiring, multiple substance metabolism is also involved in the pathogenesis of S-AKI. Future research on the therapy of S-AKI could extend towards targeting substance metabolism pathways. Moreover, our RNA sequencing data may provide more possible directions for further study on the underlying mechanisms of NLRP3 involvement in LPS-induced S-AKI.

Ferroptosis is a unique sort of regulated cell death distinguished by ferritin overaccumulation and enhanced lipid peroxidation [44,45]. Xiao et al. [21] reported the presence of ferroptosis in S-AKI using a CLP-induced animal model for the first time. Subsequently, another investigation provided consistent support in an animal model of S-AKI caused by LPS and showed that an inhibitor of ferroptosis was capable of relieving the LPS-induced S-AKI [20], which revealed that ferroptosis contributes to the development of S-AKI. An increasing number of studies have emerged in recent years demonstrating the link between NLRP3 and ferroptosis, showing that ferroptosis inhibition could mitigate NLRP3 inflammasome activation in an insecticide-induced AKI model and that NLRP3 interacts with ferroptosis in an acute lung injury model caused by sepsis [46,47]. However, the relationship between NLRP3 and ferroptosis in S-AKI remains unknown. Thus, we performed RNA sequencing of mouse renal tissues and combined it with an analysis of the FerrDb V2 database, discovering that *Nlrp3* deficiency decreased renal ferroptosis, as evidenced by the expression of ferroptosis driver genes and tissue iron detection. To the best of our knowledge, we are the first to report that NLRP3 can regulate ferroptosis in S-AKI induced by LPS, which provides a novel mechanism involving S-AKI progression by NLRP3 and indicates that targeting NLRP3 might be an effective strategy for many diseases involving ferroptosis.

There are several limitations in this study. Instead of using conditional deletion in the kidney, we utilized global *Nlrp3* deletion mice to investigate the function of NLRP3 in S-AKI. Given the expression of NLRP3 in infiltrating immune cells and renal intrinsic cells within the kidney [23], further studies using renal-specific *Nlrp3* knockout mice would help to better elucidate the effect of NLRP3 expressed in the kidney on S-AKI. In terms of mechanism exploration, we have not validated the changes in metabolic pathways discovered by RNA sequencing, and the specific mechanism by which *Nlrp3* deficiency decreased renal ferroptosis remains unclear. Future work will study the role and mechanism of NLRP3’s regulating ferroptosis and a specific metabolism pathway in S-AKI.

## 5. Conclusions

Our study verifies that NLRP3 is elevated in the kidneys of S-AKI patients and that *Nlrp3* deficiency attenuates S-AKI induced by LPS in mice. *Nlrp3* deletion reduces inflammation and ferroptosis and reverses metabolic pathway changes in the kidneys of S-AKI mice caused by LPS, which may be the underlying mechanisms for *Nlrp3* knockout in LPS-induced S-AKI. Effective treatment of S-AKI may be achieved by using NLRP3 as a therapeutic target.

## Figures and Tables

**Table 1:** The clinical data of control individuals and S-AKI patients.

Indicators	Control (n = 5)	S-AKI (n = 4)	*p* Value
Gender (number of female)	2	3	0.5238
Age (years)	29.6 ± 20.2	49.8 ± 13.6	0.1753
Serum creatinine (µmol/L)	59.1 ± 15.6	357.2 ± 311.7	0.1017
Serum urea (mmol/L) *	5.0 ± 1.2	12.2 ± 4.8	0.0252
eGFR (mL/min/1.73 m^2^) ***	114.8 ± 12.0	25.8 ± 16.4	0.0003
White blood cells (×10^9^/L)	8.6 ± 2.2	12.6 ± 4.5	0.1628
NEUT% (%)	57.4 ± 20.6	82.1 ± 5.3	0.0773
C-reactive protein (mg/L)	2.0 ± 1.6	101.0 ± 100.0	0.1039
ESR (mm/h)	57.0 ± 16.1	77.0 ± 43.0	a
Procalcitonin (ng/mL)	<0.05 #	6.8 ± 10.3	
Urinary protein (g/24 h)	5.3 ± 4.7	0.8 ± 0	a
Urine protein-to-creatinine ratio (g/g)	5.3 ± 3.7	2.1 ± 1.6	a

The data are expressed as the mean ± SD. eGFR: estimated glomerular filtration rate, determined by the Chronic Kidney Disease Epidemiology Collaboration (CKD-EPI) equation [16]. NEUT%: percentage of neutrophils among white blood cells. ESR: erythrocyte sedimentation rate. a Statistical analysis cannot be conducted as the sample size is less than three. # The result is lower than the minimum detection level. * Significant difference between two groups (*p* < 0.05). *** Significant difference between two groups (*p* < 0.001).

**Table 2:** Mouse primers for qPCR.

Gene	Primer Sequences	
β-actin	Forward	5′-CACTGTCGAGTCGCGTCC-3′
	Reverse	5′-TCATCCATGGCGAACTGGTG-3′
KIM-1	Forward	5′-TTAGGTGCTAGGAGGAGACAA-3′
	Reverse	5′-TATCACACCTGCAAATAGGACT-3′
NGAL	Forward	5′-AATTACCCTGTATGGAAGAACC-3′
	Reverse	5′-CAGAGAAGATGATGTTGTCGTC-3′
IL-6	Forward	5′-TAGTCCTTCCTACCCCAATTTCC-3′
	Reverse	5′-TTGGTCCTTAGCCACTCCTTC-3′
TNF-α	Forward	5′-CCTGTAGCCCACGTCGTAG-3′
	Reverse	5′-GGGAGTAGACAAGGTACAACCC-3′
CCL2	Forward	5′-TTAAAAACCTGGATCGGAACCAA-3′
	Reverse	5′-GCATTAGCTTCAGATTTACGGGT-3′
CCL5	Forward	5′-ATATGGCTCGGACACCACTC-3′
	Reverse	5′-ACTGCAAGATTGGAGCACTT-3′
CXCL9	Forward	5′-GGAGTTCGAGGAACCCTAGTG-3′
	Reverse	5′-GGGATTTGTAGTGGATCGTGC-3′
Hmox1	Forward	5′-AAGCCGAGAATGCTGAGTTCA-3′
	Reverse	5′-GCCGTGTAGATATGGTACAAGGA-3′
Qsox1	Forward	5′-TGGCGCTAATGTGCAGACTC-3′
	Reverse	5′-CACTGCCACAGCATGGTACT-3′
Timp1	Forward	5′-GCAACTCGGACCTGGTCATAA-3′
	Reverse	5′-CGGCCCGTGATGAGAAACT-3′
Chac1	Forward	5′-CTTGGTGGCTATGACACTAAGG-3′
	Reverse	5′-CCTCGGCAAGCAAGGATCTG-3′
Slc7a11	Forward	5′-GGCACCGTCATCGGATCAG-3′
	Reverse	5′-CTCCACAGGCAGACCAGAAAA-3′
Atf3	Forward	5′-CCTCAGAAGTCAGTGCGACC-3′
	Reverse	5′-CATCCGATGGCAGAGGTGTT-3′
Acsl4	Forward	5′-CTCACCATTATATTGCTGCCTGT-3′
	Reverse	5′-TCTCTTTGCCATAGCGTTTTTCT-3′

## Data Availability

The data presented in this study are available on request from the corresponding author.

## Supplementary Materials

The following supporting information can be downloaded at https://www.mdpi.com/article/10.3390/biology12091188/s1. Table S1: 22 ferroptosis-related DEGs (FerDEGs).
